# The Effect of Incorporating Dimethylaminohexadecyl Methacrylate and/or 2-Methacryloyloxyethyl Phosphorylcholine on Flexural Strength and Surface Hardness of Heat Polymerized and 3D-Printed Denture Base Materials

**DOI:** 10.3390/ma17184625

**Published:** 2024-09-20

**Authors:** Njood F. AlAzzam, Salwa O. Bajunaid, Heba A. Mitwalli, Bashayer H. Baras, Michael D. Weir, Hockin H. K. Xu

**Affiliations:** 1 Department of Prosthetic Sciences, College of Dentistry, King Saud University, Riyadh 60169-15, Saudi Arabia; sbajunaid@ksu.edu.sa; 2 Department of Restorative Dental Sciences, College of Dentistry, King Saud University, Riyadh 60169-15, Saudi Arabia; hmitwalli1@ksu.edu.sa (H.A.M.);; 3 Department of Advanced Oral Sciences and Therapeutics, University of Maryland School of Dentistry, Baltimore, MD 21201, USA

**Keywords:** denture base material, removable dentures, denture stomatitis, candida albicans, antifungal, flexural strength, surface hardness

## Abstract

Background: A major disadvantage of polymethyl methacrylate (PMMA) acrylic resins is susceptibility to biofilm accumulation. The incorporation of antimicrobial agents is a reliable prevention technique. The purpose of this study is to investigate the effect of incorporating dimethylaminohexadecyl methacrylate (DMAHDM) and/or 2-methacryloyloxyethyl phosphorylcholine (MPC) into heat-polymerized (HP) and 3D-printed (3DP) denture base materials on the flexural strength, modulus of elasticity, and surface hardness. Methods: DMAHDM and/or MPC were mixed with the acrylic resin liquid of a heat-polymerized (ProBase Hot) and a 3D printed (NextDent Denture 3D) material at mass fractions of 1.5% and 3% and a combination of 3% MPC and 1.5% DMAHDM. Results: Significant differences in mechanical properties between the control and experimental groups have been detected (*p*-value < 0.0001). In HP materials, the addition of DMAHDM and/or MPC generally decreased the flexural strength, from (151.18 MPa) in G1 down to (62.67 MPa) in G5, and surface hardness, from (18.05 N/mm^2^) down to (10.07 N/mm^2^) in G5. Conversely, in 3DP materials, flexural strength was slightly enhanced, from (58.22 MPa) in G1 up to (62.76 MPa) in G6, although surface hardness was consistently reduced, from (13.57 N/mm^2^) down to (5.29 N/mm^2^) in G5. Conclusion: It is recommended to carefully optimize the concentrations of DMAHDM and/or MPC to maintain mechanical integrity.

## 1. Introduction

Denture base acrylic resins can be categorized according to their manufacturing technique into auto-polymerized acrylic resins, heat-polymerized acrylic resins, light and microwave polymerized acrylic resins, and computer-aided design/computer-aided manufactured (CAD/CAM) acrylic resins [1,2,3,4,5]. Acrylic resin is the optimum material for the fabrication of the removable denture base, as it has fulfilled most of the requirements of an ideal removable denture base material [6,7]. It has multiple advantages including favorable working characteristics, ease of processing, accurate fit and stability, good color stability, superior esthetics, lower cost, biocompatibility, and reliability [7,8]. However, its major disadvantage is its susceptibility to biofilm accumulation, such as candida albicans, as it favors pathogen colonization and acts as a pathogen reservoir [7,9,10,11]. Mechanical attachment of biofilm to acrylic resin surface may be attributed to some local factors such as porosities, surface roughness, poor denture hygiene, and continuous denture use [12,13,14]. Continuous denture use, particularly without removing them at night, can lead to inflammation primarily due to several factors related to mechanical stress ending in mechanical trauma, and microbial growth of candida albicans due to reduced salivary flow to the area and improper cleansing of the denture [12,13,14]. Other disadvantages of acrylic resins include the leakage of toxic monomers and the release of microparticles leading to possible toxicity [15]. Conventionally, heat-polymerized acrylic resins were the dominant denture base material; they are well known for their strength, dimensional stability, and long-term performance. These are mainly polymethylmethacrylate (PMMA)-based and processed by mixing a liquid monomer and a powdered polymer; the mix is then subjected to heat to initiate the polymerization process [16,17]. Recently, 3D printing technology has been evolving rapidly, and dentistry is no exception. A 3D-printed acrylic resin is also composed of a polymethylmethacrylate (PMMA)-based light-curable resin. The additive technique (3D printing) is used for the manufacturing of removable denture bases and removable partial denture frameworks [18,19]. The process involves digital designing, a layer-by-layer deposition of the resin, and selective curing using a light source. The technique showed promising results with faster production, fewer clinical visits, easier modifications, high precision, and greater accuracy [17,18].

Denture stomatitis (DS) is defined as an inflammatory reaction of the edentulous mucosa underlying removable dentures. This inflammatory reaction is common, with a reported prevalence of from 15% to 70% of denture wearers [12]. There are several linked causative and contributing factors of such a condition, including poor denture and oral hygiene, poor denture quality, and continuous denture use [20,21]. DS is most associated with the presence of candida albicans on the denture fitting surface and oral mucosa [22,23]. Chemical and mechanical methodologies have been introduced to maintain the hygiene of removable prostheses [24]. A proven way to control and eliminate these pathogenic processes is to achieve a more durable material by incorporating some antimicrobial agents within the dental material [25].

**Figure 1:**
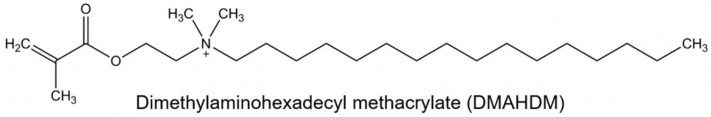
The structure of DMAHDM shows a chain length of 16 units.

Efforts were made to enhance acrylic resins’ antimicrobial properties [26,27,28,29,30,31,32]. These attempts include microbial repelling multilayer coating [33], incorporating various metal oxide fillers and fibers [30,34,35,36], embedding spherical Ag NPs [31,37,38], addition of zinc oxide nanoparticles (ZnO NPs) [29,32], reinforcement with nanodiamonds (ND) [30], plant extracts and phyto-compounds [39], and mesoporous silica coated with cerium oxide nanoparticles [40]. Quaternary ammonium methacrylates (QAMs) are antimicrobial monomers that are stabilized to the dental resin and act as immobilized bactericides which have led to promising antimicrobial results [41,42,43,44,45,46]. They immobilize the antimicrobial components in the resin matrix by covalent bond formation, which then acts as a free bactericide [41,42,43,44,45,46]. The QAM, with an alkyl chain length of 16 units, is referred to as dimethylaminohexadecyl methacrylate (DMAHDM) (Figure 1) and it has shown strong antimicrobial potential when copolymerized with dental resins [47,48]. Dimethylaminohexadecyl methacrylate (DMAHDM) is a powerful antimicrobial agent used in dental materials, primarily due to its quaternary ammonium structure, which is responsible for its broad-spectrum antimicrobial activity. The mechanism of action of DMAHDM centers on its ability to disrupt bacterial cell membranes. The quaternary ammonium group in DMAHDM is positively charged, which allows it to interact with and penetrate the negatively charged bacterial cell membranes. This interaction destabilizes the membrane, leading to leakage of cellular contents and ultimately causing bacterial cell death. Additionally, the long alkyl chain in DMAHDM enhances its membrane-disruptive capabilities, making it particularly effective against a wide range of bacteria. Besides killing bacteria, DMAHDM also inhibits biofilm formation by preventing bacterial adhesion to surfaces, which is crucial for reducing persistent infections [41,42,43,44,45,46,47,48].

The monomer 2-Methacryloyloxyethyl phosphorylcholine (MPC) is a biocompatible monomer widely used in biomedical applications due to its ability to form a protective hydration layer on surfaces, which effectively resists protein adsorption and reduces microbial adhesion. The phosphorylcholine group in MPC mimics the structure of cell membrane phospholipids, allowing it to tightly bind water molecules and create a hydrophilic barrier. This barrier prevents proteins and other biomolecules from adhering to the surface, which in turn reduces the likelihood of microbial biofilm formation, a critical factor in preventing infections on medical and dental devices. MPC’s protein-repellent properties are due to its zwitterionic nature, which repels charged species and maintains surface cleanliness, thus enhancing the biocompatibility and longevity of the materials it is incorporated into. This mechanism is particularly beneficial in applications such as dental resins [49,50,51]. MPC-modified denture base materials significantly reduce the adhesion of proteins and bacteria, leading to a lower incidence of infections [51]. Dental composites containing MPC showed a substantial reduction in bacterial adhesion compared to unmodified materials, which translates to improved oral health outcomes for patients wearing dentures [51]. Additionally, the potential of MPC to maintain the mechanical properties of the denture base while enhancing its resistance to microbial colonization, ensuring both durability and functionality, has been previously established [52]. The development of new dental materials that have the potential to repel proteins, inhibit their adsorption, and prevent bacterial adhesion is highly beneficial to the dental industry, but careful evaluation of the effects on the mechanical properties should be kept in mind.

To date, there have been few reports on the effect of incorporating DMAHDM and/or MPC into heat-cured, auto-cured, reline self-cured acrylic resins and other dental materials; however, to the best of the authors’ knowledge, none have been conducted on 3D printed acrylic resin materials. The purpose of this study is to investigate the effect of incorporating DMAHDM and/or into heat-polymerized (ProBase Hot) and 3D printed (NextDent Denture 3D) denture base resins on the flexural strength, modulus of elasticity, and surface hardness of the produced material. The hypothesis to be tested is whether the incorporation of DMAHDM and/or MPC into a heat-polymerized (ProBase Hot) and 3D printed (NextDent Denture 3D) denture base resin would or would not compromise the flexural strength, modulus of elasticity, and surface hardness when compared to the control counterparts without the addition of DMAHDM and/or MPC. Although the main focus of this study was to investigate whether the addition of DMAHDM and/or MPC would adversely affect the mechanical properties of denture base materials, future studies will focus on the antimicrobial effects of DMAHDM and/or MPC when tested against oral biofilms causing denture stomatitis.

## 2. Materials and Methods

### 2.1. Incorporation of DMAHDM into the Acrylic Resin Liquid

**Figure 2:**
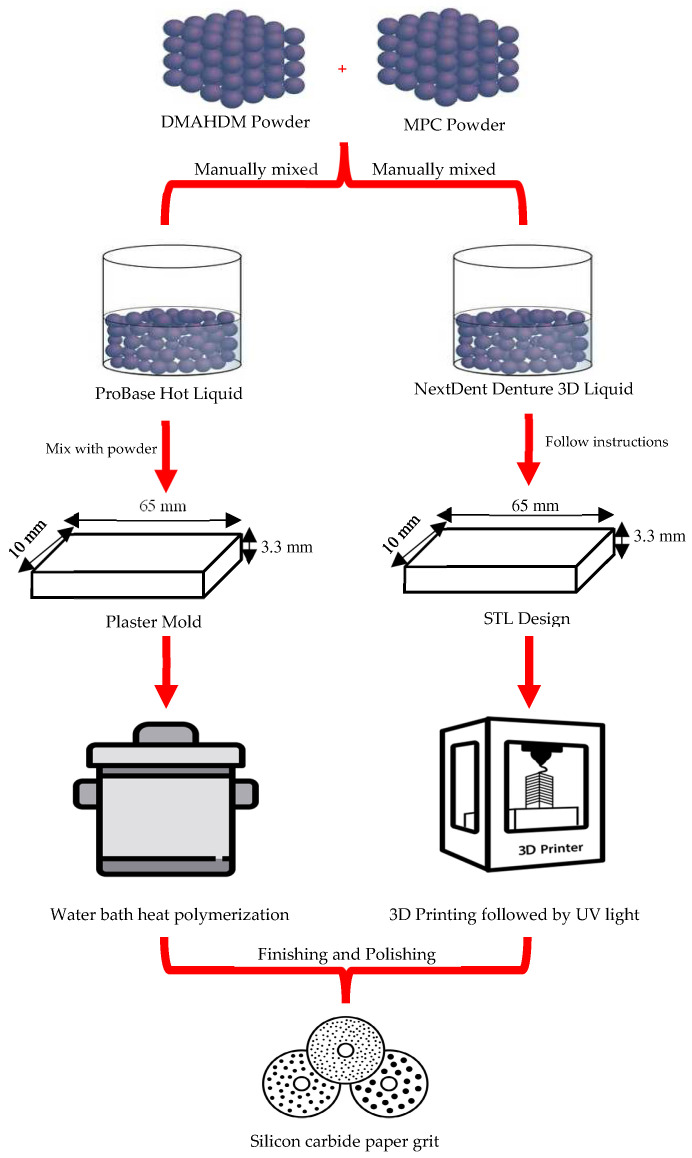
Graphical presentation of the specimens’ formulation procedure.

DMAHDM was synthesized using a modified Menschutkin reaction method [45,53,54,55]. This reaction is carried out between the organo-halide and the tertiary amine [56]. Briefly, a combination of 10 mmol of 2-(dimethylamino) ethyl methacrylate (DMAEMA, Sigma-Aldrich, St. Louis, MO, USA, 2867-47-2) and 10 mmol of 1-bromohexadecane (BHD, TCI America, Portland, OR, USA, 112-82-3) along with 3 g of ethanol was carried out in a 20 mL clear glass vial. The reaction between Dimethylaminoethyl methacrylate (DMAEMA) and 1-bromohexadecane results in the formation of a quaternary ammonium salt [57,58,59]. In the ^1^*H* NMR spectra, chemical shifts corresponding to the aliphatic protons from the hexadecyl chain (typically from around 0.85 to 1.4 ppm for the CH_2_ and CH_3_ groups) would be observed, the methylene protons adjacent to the ester and nitrogen groups, and the methyl groups attached to the quaternary nitrogen appearing as a singlet around 3.0–3.3 ppm [57,58,59]. The ^13^*C* NMR spectra would show peaks for the carbonyl group, aliphatic carbons, and quaternary ammonium methyl groups [57,58,59]. The IR spectra would display strong absorption bands for the ester carbonyl group around 1720–1740 cm^−1^, aliphatic C-H stretching around 2850–2950 cm^−1^, and C-N stretching in the range of 900–1200 cm^−1^, confirming the formation of the quaternary ammonium salt [57,58,59]. The reaction then proceeded for 24 h at 70 °C. After that, the solvent was evaporated under a vacuum, leaving DMAHDM as a waxy solid [48]. DMAHDM was manually mixed with the acrylic resin liquid at mass fractions of 1.5% and 3%, respectively. MMA with 0% DMAHDM will be used as the control group for comparison (Figure 2).

### 2.2. Incorporation of MPC into Acrylic Resin Liquid

MPC was commercially purchased from Sigma-Aldrich (MPC, Sigma-Aldrich, St. Louis, MO, USA, 67881-98-5). The MPC powder was manually mixed with the acrylic resin liquid at a mass fraction of 1.5% and 3%. Acrylic resin liquid with 0% MPC will be used as the control group for comparison [53,54] (Figure 2).

For the acrylic resin liquid that contained both DMAHDM and MPC, the mass fractions of these materials were 1.5% and 3%, respectively. As suggested by previous studies, the mentioned masses of the DMAHDM and MPC had led to a greater reduction in biofilm growth without compromising the mechanical or physical properties compared to using DMAHDM or MPC alone [54,60]. After the complete mixing of DMAHDM and MPC into the acrylic resin liquid, the combined liquid was then mixed with the resin powder (heat-polymerized ProBase Hot or 3D-printed NextDent Denture 3D) to fabricate the specimens [53].

### 2.3. Preparation of the Testing Groups

**Figure 3:**
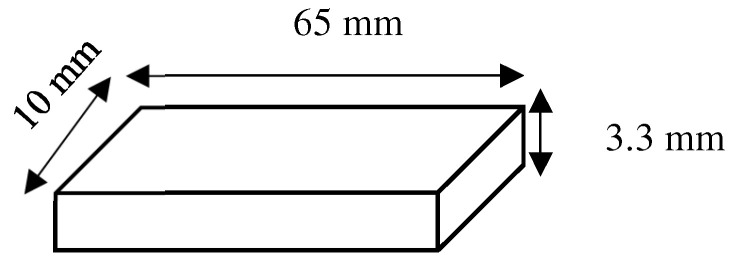
Rectangular specimens design measuring 65 × 10 × 3.3 mm (±0.2 mm).

The heat-polymerized denture base material (ProBase Hot, Ivoclar Vivadent Inc., Schaan, Liechtenstein) was selected to be the HP group, while the 3D-printed denture base material (NextDent Denture 3D, NextDent, Soesterberg, The Netherlands) was selected for the 3DP group. Material specifications, as noted by the manufacturers, are mentioned in Table 1. Specimens were then fabricated as rectangular specimens measuring 65 × 10 × 3.3 mm (±0.2 mm) (Figure 3).

#### 2.3.1. Heat-Polymerized (HP) Denture Base Group

The heat-polymerized denture base material (ProBase Hot, Ivoclar Vivadent Inc., Schaan, Liechtenstein) was mixed according to the manufacturer’s instructions in a ratio of 22.5 g polymer (powder): 10 mL monomer (liquid previously mixed with DMAHDM and/or MPC) and left in a closed mixing cup at room temperature for 8–10 min. The monomer used in this ratio is the one mixed with DMAHDM and/or MPC. The paste was then pressed into previously fabricated custom plaster molds (rectangular specimens measuring 65 × 10 × 3.3 mm (±0.2 mm) Figure 3) and placed inside flasks. The flasks were closed with a load of 80 bar pressure and fixed with clamps to maintain the pressure. Heat-polymerization was carried out by placing the flasks in a cold-water bath, which was heated up to 100 °C/212 °F and boiled for 45 min. The flasks were then left to cool at room temperature for 30 min, followed by complete cooling with cold water. Specimens were then finished using silicon carbide paper grit P1200 (Paper SiC P1200; Struers GmbH, Ballerup, Denmark), according to ISO 20795-1 [61] (Figure 2). Final dimensions were measured and confirmed using a digital caliper. After finishing, all specimens were stored for 24 h in 37 °C distilled water before testing [61]. The specimens were divided into six groups, with ten specimens in each group, as described below (N = 60):

(1) ProBase Hot control; “Control HP” (*n* = 10);
(2) ProBase Hot + 1.5% MPC; “1.5% MPC HP” (*n* = 10);
(3) ProBase Hot + 3% MPC; “3% MPC HP” (*n* = 10);
(4) ProBase Hot + 1.5% DMAHDM; “1.5% DMAHDM HP” (*n* = 10);
(5) ProBase Hot + 3% DMAHDM; “3% DMAHDM HP” (*n* = 10);
(6) ProBase Hot + 3% MPC + 1.5% DMAHDM; “3% MPC + 1.5% DMAHDM HP” (*n* = 10).

#### 2.3.2. D-Printed (3DP) Denture Base

Specimens were designed as rectangular specimens measuring 65 × 10 × 3.3 mm (±0.2 mm) and saved as a standard tessellation language (STL) file, then fabricated using a 3D printing machine (DentalFab, Microlay Dental 3D Printers, Madrid, Spain) at a 45-degree angle with a supporting structure. After manually mixing the DMAHDM and/or MPC into the liquid resin, the bottles containing the liquid resin were shaken manually for 5 min and then mixed for 2.5 h using a mixer (LC-3DMixer, NextDent, Soesterberg, The Netherlands) before being poured into the resin tray as instructed by the manufacturer. The mixture was stirred in the tray for 30 s with a plastic scraper before printing. Following printing, the specimens were cleaned with 90% isopropyl alcohol for 5 min and polymerized from all sides for 30 min using ultraviolet light (385 nm) with a UV-A type 3 post-polymerization lightbox (LC3DPrint Box NextDent, Soesterberg, The Netherlands). Specimens were then finished using silicon carbide paper grit P1200 (Paper SiC P1200; Struers GmbH, Ballerup, Denmark), according to ISO 20795-1 [61] (Figure 2). Final dimensions were measured and confirmed using a digital caliper. After finishing, all specimens were stored for 24 h in 37 °C water before testing [61]. The specimens were divided into six groups, with ten specimens in each group, as described below (N = 60):

(1) NextDent Denture 3D + control; “Control 3DP” (*n* = 10);
(2) NextDent Denture 3D + 1.5% MPC; “1.5% MPC 3DP” (*n* = 10);
(3) NextDent Denture 3D + 3% MPC; “3% MPC 3DP” (*n* = 10);
(4) NextDent Denture 3D + 1.5% DMAHDM; “1.5% DMAHDM 3DP” (*n* = 10);
(5) NextDent Denture 3D + 3% DMAHDM; “3% DMAHDM 3DP” (*n* = 10);
(6) NextDent Denture 3D + 3% MPC + 1.5% DMAHDM; “3% MPC + 1.5% DMAHDM 3DP” (*n* = 10).

### 2.4. Sample Size

The sample size was determined using the power package in R software (R package version 1.3-0, R Core Team 2022, R Foundation for Statistical Computing, Vienna, Austria) using flexural strength as the main outcome for the calculation; we used eight samples per group to detect an effect size of 0.55 (f) with 0.80 power at alpha = 0.05. The sample size was, however, increased to 10 to compensate for any specimen damage or loss during the experiments, which, when maintained, was able to detect an effect size of 0.5 (f) with a 0.82 power instead.

### 2.5. Randomization and Blinding

A randomization design was followed where the specimens of each group were encoded with a random number from 1 to 60 by using a research randomization software (Research Randomizer, Version 4.0, Social Psychology Network, Middletown, CT, USA) [62]. The data sheet of the numbering acquired from the research randomization software was saved and kept aside in a password-secured Excel sheet. The specimens were then arranged in a sequential arrangement before testing. After all tests were performed, the numbering sheet was revealed, and the data were arranged by the specimens’ groups.

### 2.6. Flexural Strength

**Figure 4:**
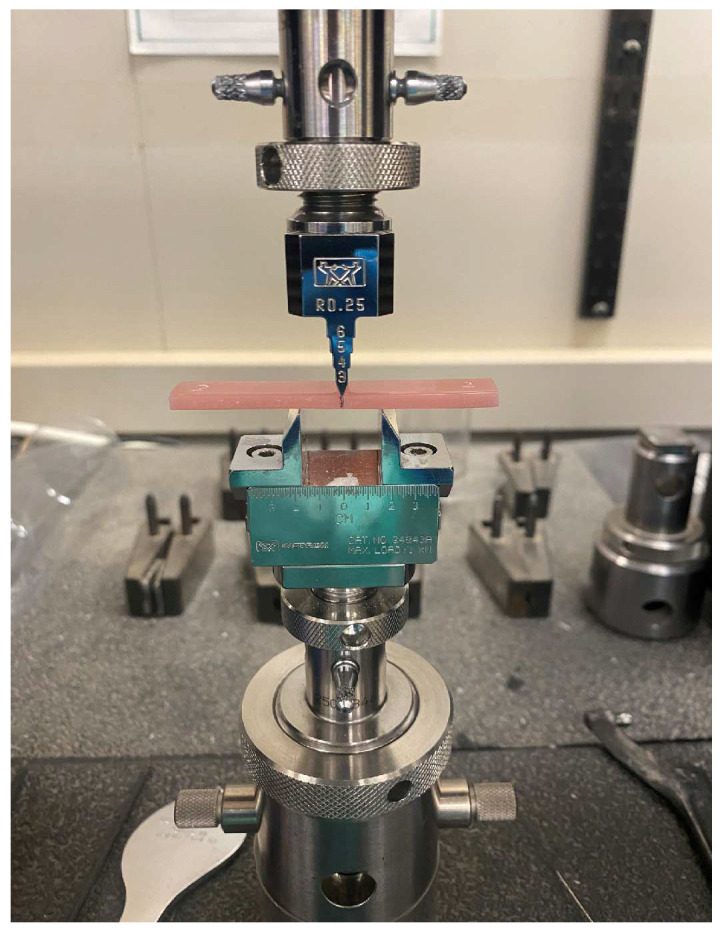
Specimen fixed on the INSTRON machine, where the middle of the specimen is marked by a black line to ensure proper placement.

A 3-point bend test was performed by a universal testing machine (5965 Universal Testing System, INSTRON, Norwood, MA, USA), after calibration of the machine according to the manufacturer’s instructions, it was set to a 15 mm span at a crosshead speed of 1 mm/min. The specimen’s midsection was marked by a black marker to help orient the specimen to the machine. Then, it was fixed on the machine (Figure 4), and the flexural strength readings were obtained when the load was conducted to the specimen until visual failure (fracture) was reached and the flexural strength was then calculated using the following formula:

$$S=\frac{3PmaxL}{2bh^{2}}$$

where *Pmax* is the maximum load on the load-displacement curve, *L* is the flexure span, *b* is the specimen width, and *h* is the specimen thickness [53].

### 2.7. Surface Hardness

**Figure 5:**
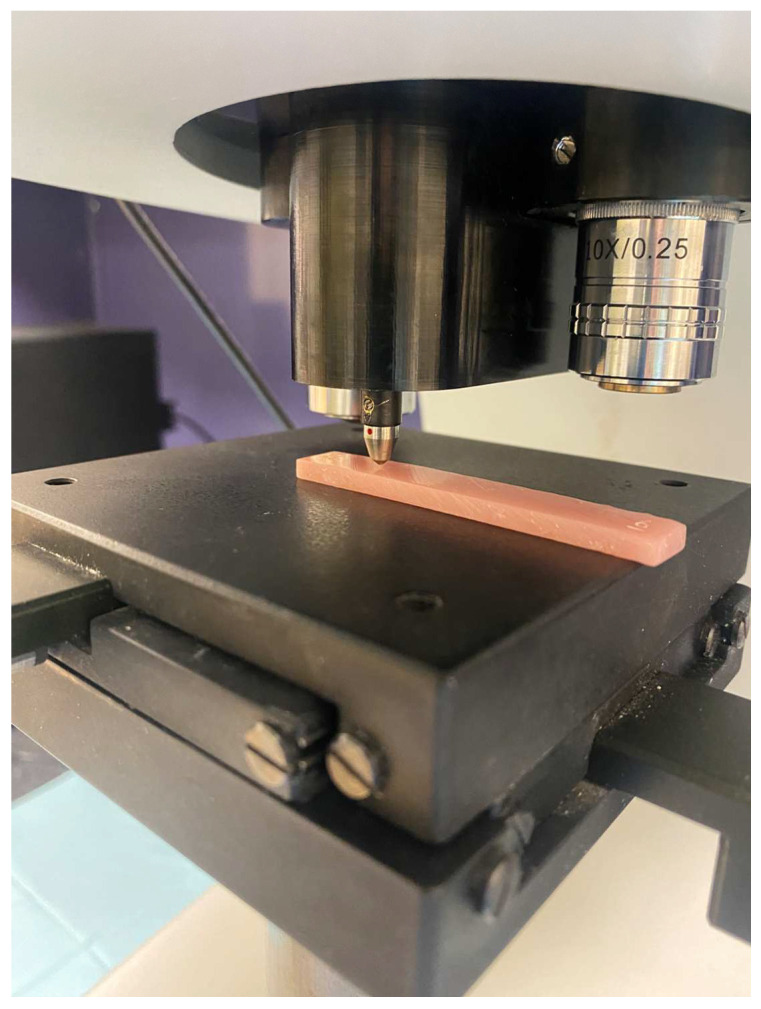
A square-based pyramid diamond indenter performing the Vickers hardness test on the rectangular specimen.

A Vickers hardness testing device (Duramin-5; Struers, Ballerup, Denmark) was calibrated according to the manufacturer’s instructions and was used to perform the surface microhardness test on randomly selected regions of the specimens at a force of 1.96 N for 15 s. An indentation was created with a square-based pyramid diamond indenter (Figure 5). The diagonals of the pyramid impressed on the specimen were measured on a microscopic scale by the eyepiece operator of the testing machine, and then the surface microhardness (VHN) was calculated using the following formula:

$$H=0.1891\frac{F}{d^{2}}$$

where *H* is Vickers hardness (VHN); *F* is the force in newtons (N); and *d* is the mean length of the two diagonals in millimeters (mm) [63].

### 2.8. Statistical Analysis

Statistical analysis was performed with SPSS Statistics v.20 (IBM, Endicott, Armonk, NY, USA) at α = 0.05. Two independent variables were assessed: the two types of acrylic materials, the DMAHDM and/or MPC-incorporated acrylic materials. A multivariant analysis of variance (MANOVA) test was performed to compare the mean differences between the groups.

## 3. Results

### 3.1. Flexural Strength

**Figure 6:**
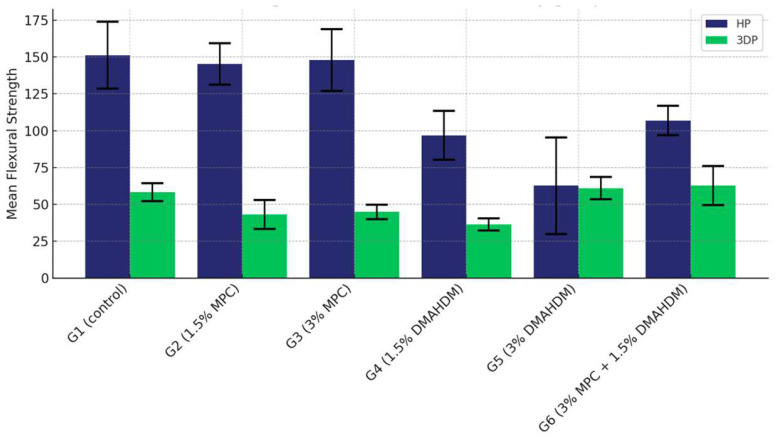
Comparison of the mean flexural strength values between the six study groups in each of the tested materials (HP, 3DP). A highly statistically significant difference is detected among the six groups of the two study materials, and a notable superiority of the HP material is noticed.

The comparison of the mean flexural strength values among the six study groups of the two materials (G1 (control), G2 (1.5% MPC), G3 (3% MPC), G4 (1.5% DMAHDM), G5 (3% DMAHDM), and G6 (1.5% DMAHDM + 3% MPC)) are presented in Figure 6 and Table 2. The one-way analysis of variance test shows a highly statistically significant difference in the mean flexural strength values among the six study groups (*p* < 0.0001).

The comparison of the mean flexural strength values among the six study groups in the HP material shows that Group G3 (3% MPC) demonstrated the highest flexural strength, closely followed by Group G2 (1.5% MPC). The control group, G1, displayed moderately high values, surpassing those of Groups G4 (1.5% DMAHDM), G6 (1.5% DMAHDM + 3% MPC), and G5 (3% DMAHDM), with G5 showing the lowest strength in this material (Table 3).

Conversely, when examining the 3DP material, Group G5 (3% DMAHDM) rose to the top with the highest strength, with G6 (1.5% DMAHDM + 3% MPC) trailing slightly behind. The control group maintained a middle position, performing better than G2 (1.5% MPC), G3 (3% MPC), and notably higher than G4 (1.5% DMAHDM), which had the lowest strength within the 3DP groups (Table 4).

**Figure 7:**
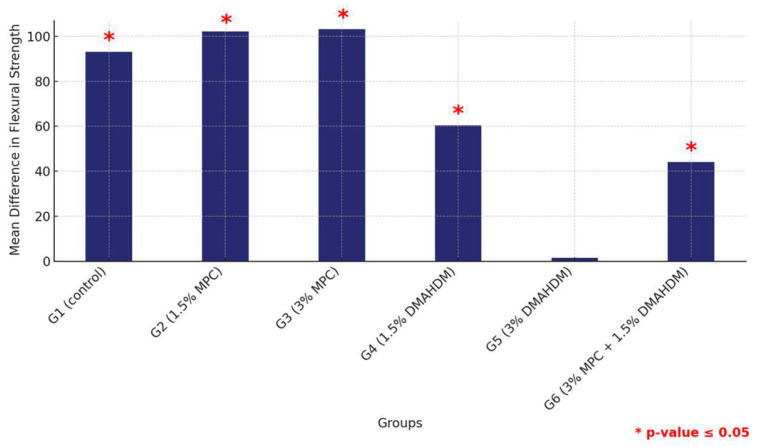
Comparison of mean flexural strength differences between the six study groups between the two tested materials. Displaying significant differences among all groups except G5. Significant comparisons are marked with a red star (* = *p* = value ≤ 0.05).

The comparative analysis between the two materials revealed a consistent pattern where all groups except for G5 (3% DMAHDM) exhibited significantly higher flexural strength in the HP material compared to their counterparts in 3DP, all marked by statistically significant differences (*p* < 0.0001). This comprehensive assessment underscores the material-dependent performance variations within the study groups, highlighting the superior structural integrity offered by the HP material across multiple groups (Figure 7 and Table 5).

### 3.2. Elastic Modulus

**Figure 8:**
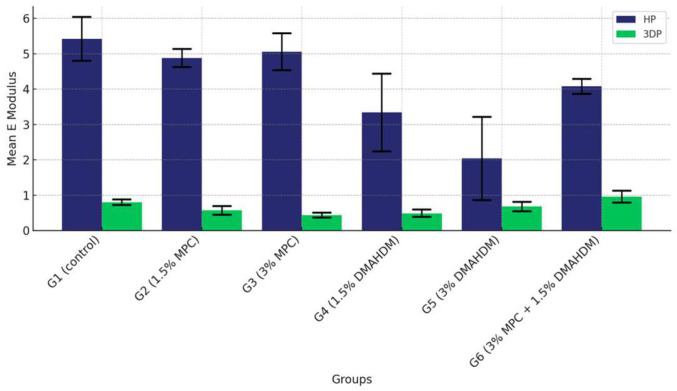
Comparison of the mean elastic modulus values between the six study groups in each of the tested materials (HP, 3DP). A highly statistically significant difference is detected among the six groups of the two study materials, showing a noticeable superiority of the HP material over the 3DP.

The comparison of the mean elastic modulus values among the six study groups of the two study materials (G1 (control), G2 (1.5% MPC), G3 (3% MPC), G4 (1.5% DMAHDM), G5 (3% DMAHDM) and G6 (1.5% DMAHDM + 3% MPC)) is given in Figure 8 and Table 6. The one-way analysis of variance shows a highly statistically significant difference in the mean elastic modulus values among the six study groups (*p* < 0.0001) in both materials.

In the post-hoc analysis of the pair-wise comparison of mean elastic modulus values among the six study groups in the HP material, Group G1 (control) exhibited the highest elastic modulus. Groups G2 (1.5% MPC) and G3 (3% MPC) displayed superior modulus values, followed by G4 (1.5% DMAHDM), which registered higher values than G5 (3% DMAHDM), which, in turn, was surpassed by G6 (1.5% DMAHDM + 3% MPC), indicating a nuanced hierarchy within the lower-performing groups (Table 7).

In contrast, with the 3DP material, G1 (Control) again topped the chart. G6 (1.5% DMAHDM + 3% MPC) emerged as the leader among the non-control groups, significantly exceeding the others, followed by G5 (3% DMAHDM), which also performed robustly, showing higher modulus values than the remaining groups. This pattern underscores the varied responses of the groups under different material conditions (Table 8).

**Figure 9:**
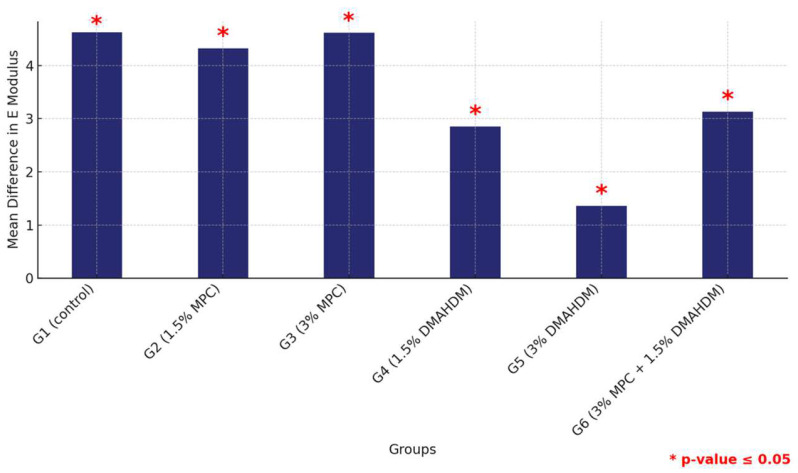
Comparison of mean elastic modulus differences between the six study groups between the two tested materials. Displaying significant differences among all groups. Significant comparisons are marked with a red star (* = *p* = value ≤ 0.05).

Furthermore, a comprehensive comparison of the mean elastic modulus values between the HP and 3DP materials across all groups revealed consistently higher values for the HP material, confirming its superior mechanical properties with significant statistical backing, thereby illustrating the material-dependent variations in mechanical robustness within the study groups (Figure 9 and Table 9).

### 3.3. Surface Hardness

**Figure 10:**
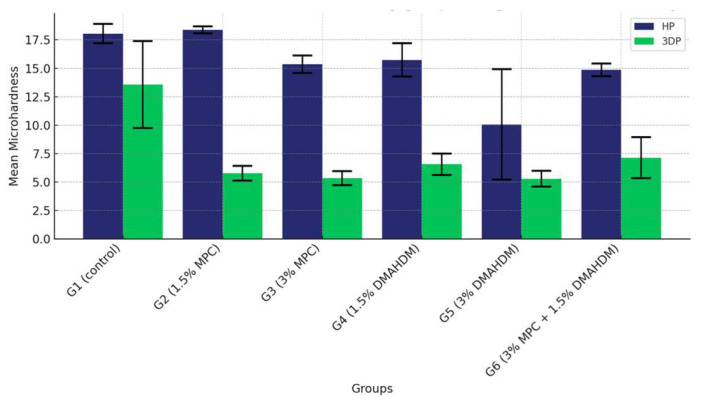
Comparison of the mean surface hardness values between the six study groups in each of the tested materials (HP, 3DP). A highly statistically significant difference is detected among the six groups of the two study materials, showing a noticeable superiority of the HP material over the 3DP material.

The comparison of the mean surface hardness values among the six study groups of the two study materials (G1 (control), G2 (1.5% MPC), G3 (3% MPC), G4 (1.5% DMAHDM), G5 (3% DMAHDM), and G6 (1.5% DMAHDM + 3% MPC)) is given in Figure 10 and Table 10. The one-way analysis of variance shows a highly statistically significant difference in the mean microhardness values among the six study groups (*p* < 0.0001).

**Figure 11:**
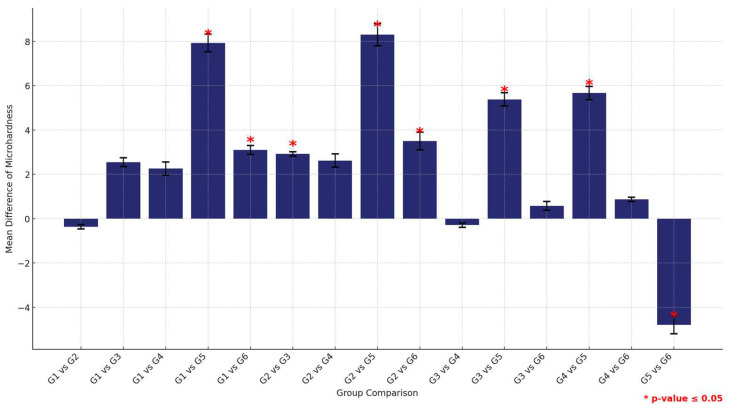
Multiple comparisons of mean surface hardness values among the six study groups using the HP material. Significant comparisons are marked with a red star (* = *p* = value ≤ 0.05).

The post-hoc analysis of pair-wise comparison of the mean microhardness values among the six study groups within the HP material group revealed that the control group (G1) exhibited the highest microhardness values, followed by G2 (1.5% MPC), which displayed higher microhardness than the others, positioning it as a top performer. Following closely was G3 (3% MPC) and then G4 (1.5% DMAHDM). Notably, G6 (1.5% DMAHDM + 3% MPC) demonstrated significantly higher microhardness compared to G5 (3% DMAHDM), indicating a marked difference between the lowest performers (Figure 11 and Table 11).

**Figure 12:**
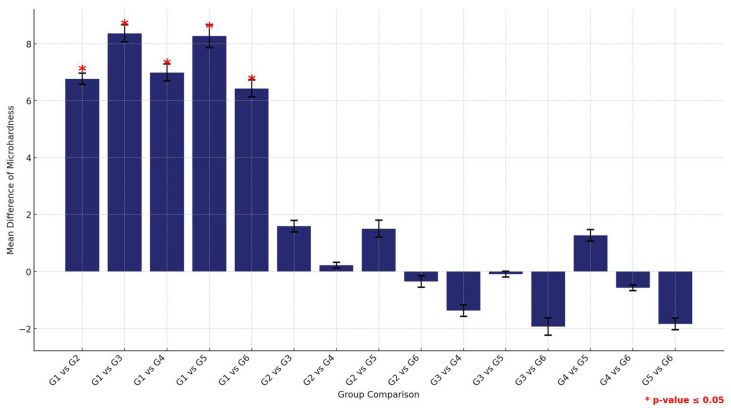
Multiple comparisons of mean surface hardness values among the six study groups using the 3DP material. Significant comparisons are marked with a red star (* = *p* = value ≤ 0.05).

In contrast, the analysis using the 3DP material showed that the control group (G1) significantly outperformed all other groups, with no other significant differences observed among the remaining groups, suggesting a more uniform performance within this material type (Figure 12 and Table 12).

**Figure 13:**
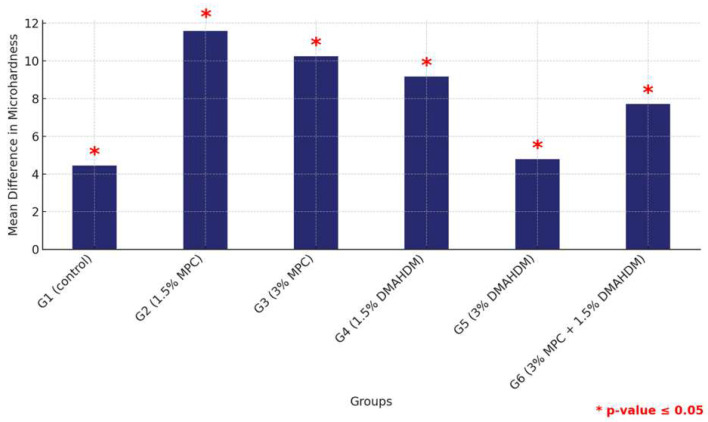
Comparison of mean surface hardness differences between the six study groups between the two tested materials. Significant comparisons are marked with a red star (* = *p* = value ≤ 0.05).

Furthermore, a comprehensive comparison between the two materials across all groups revealed that HP consistently provided higher microhardness values than 3DP. This was statistically significant across all groups, underscoring the material’s superior hardness properties. This analysis not only highlights the comparative robustness of HP over 3DP but also delineates the performance hierarchy within each material type, with the control groups consistently showing superior properties (Figure 13 and Table 13)

## 4. Discussion

This study aimed to investigate the effect of incorporating Dimethylaminohexadecyl Methacrylate (DMAHDM) and/or 2-Methacryloyloxyethyl Phosphorylcholine (MPC) into heat-polymerized (HP) and 3D-printed (3DP) denture base resin materials on flexural strength, modulus of elasticity, and surface hardness on the final material. The results revealed significant impacts on these mechanical properties among the different groups of each material and between the two types of materials. The effects varied depending on the type and the concentration of the incorporated agents, leading to the rejection of the null hypothesis and the acceptance of the hypothesis that incorporating DMAHDM and/or MPC into heat-polymerized and 3D-printed denture base materials affects flexural strength, modulus of elasticity, and surface hardness.

The incorporation of DMAHDM and/or MPC into denture base resin materials influenced the flexural strength of both the HP and 3DP materials. The control groups for both heat-polymerized (HP) and 3D-printed (3DP) materials generally exhibited higher flexural strength compared to the other study groups. For the HP materials, the control group exhibited higher flexural strength compared to the groups with 1.5% DMAHDM, 3% DMAHDM, and the combination of 1.5% DMAHDM and 3% MPC. This is consistent with the findings of previous studies that indicated a decrease in the flexural strength of heat-polymerized denture base materials with the addition of DMAHDM, attributed to potential plasticization effects and interference with the polymer matrix integrity [53,54,60,64,65]. Moreover, those studies emphasized the effect of such additives on the reduction of candidal adhesion with varying effects on flexural strength, suggesting that the incorporation of such additives should be made with caution and that further research is needed to optimize the optimum concentration. Authors should discuss the results and how they can be interpreted from the perspective of previous studies and the working hypotheses. The findings and their implications should be discussed in the broadest context possible. Future research directions may also be highlighted.

Notably, 3DP materials showed a different pattern where the addition of DMAHDM and/or MPC generally maintained or slightly improved the flexural properties. This discrepancy could be due to the differences in the fabrication processes and the enhanced interaction of these agents with the resin matrix during the photopolymerization process unique to 3D printing. Although investigating the incorporation of DMAHDM and/or MPC into 3D-printed denture base material has not been extensively performed in the literature, the addition of different additives such as zirconia, aluminum nitride, barium titanate, titanium dioxide, and graphene and silver nanoparticles had a similar effect of increasing the flexural strength of 3D-printed denture base materials [66,67].

In this study, the comparison of the 3DP material with the HP material had shown that the prior has noticeable decreased performance regarding strength. This fact was in coherence with the current literature, where HP denture base materials exhibit noticeably superior strength in comparison to 3DP materials [68,69]. It has been shown, in the literature, that alteration of conditions during polymerization reveals a significant effect on the mechanical properties of resinous materials. Mechanical properties tend to increase with an increase in temperature [70]. However, several advantages are associated with 3D-printed denture bases that make them an increasingly viable alternative. One of the major advantages connected with 3DP is its speed of fabrication. Dentures could be constructed at a much faster rate compared to the traditional, time-consuming heat-curing process [68]. It offers a high degree of accuracy and personalization in the creation of dentures with ultra-accuracy and fit directly from a digital design, helping to minimize the need for adjustments and remakes [68,69]. Due to the digital workflow, this procedure facilitates the process of manufacturing, avoiding the physical molds of traditional methods and making easy storage and modification of designs for their subsequent use [68]. It is more environmentally friendly and cost-effective with less material wastage involved in 3D printing [68]. The results of this study suggest that the addition of DMAHDM and/or MPC can even enhance the strength of the 3DP which can be an additional benefit in such a material; however, it is important to notice that even though the HP material was negatively affected by this addition, it still maintained superior strength in comparison to the 3DP.

Similarly, the modulus of elasticity was affected by the incorporation of DMAHDM and/or MPC. The control groups maintained higher modulus values, suggesting that the addition of these agents may compromise the rigidity of the material. This indicates that the addition of DMAHDM and/or MPC reduces the rigidity of both HP and 3DP resins, with a more pronounced effect observed in the latter. This observation is consistent with previous studies, which found that adding DMAHDM and/or MPC to denture base materials often reduces their modulus of elasticity due to alterations in the polymer matrix structure [53,64,71].

The surface hardness results indicated that both DMAHDM and/or MPC, particularly at higher concentrations, significantly reduced the hardness of both denture base materials. This observation aligns with the existing literature, where the addition of antimicrobial monomers to dental resins could reduce surface hardness, possibly due to the plasticizing effect of the monomers and the disruption of the polymer network [71,72].

Previous studies have highlighted the dual benefits of incorporating DMAHDM and/or MPC into dental resins for their antimicrobial and protein-repellent properties. The current study supports these findings, showing that these agents did not severely compromise the mechanical properties of the denture base resins while potentially offering enhanced antimicrobial benefits. For instance, several studies have demonstrated that the incorporation of quaternary ammonium compounds like DMAHDM and protein-repellent agents such as MPC can significantly reduce biofilm formation without drastically affecting the resin’s mechanical properties [64,71,73,74].

The findings from this research project have significant clinical implications for the field of prosthodontics. The incorporation of DMAHDM and/or MPC into denture base materials presents a promising approach to enhancing the antimicrobial properties and longevity of removable dentures, addressing common issues such as denture stomatitis and biofilm formation. By improving the flexural strength and modulus of elasticity, particularly in 3D-printed materials, these modifications can lead to more durable and resilient dentures, providing better comfort and functionality for the patients. Additionally, the reduced surface hardness observed, especially in 3DP materials, suggests the need for careful consideration of the concentrations used to balance antimicrobial efficacy with mechanical integrity. Overall, this research supports the development of advanced denture materials that can improve oral health outcomes, reduce the incidence of denture-related infections, and enhance the overall quality of life for denture wearers.

This research has some limitations that should be acknowledged. Firstly, this study was conducted in vitro, which may not fully replicate the complex oral environment where factors such as saliva, varying pH levels, and mechanical stresses play crucial roles. The long-term effects of incorporating DMAHDM and/or MPC into denture base materials were not assessed, leaving questions about the durability and stability of these modifications over time unanswered. Further research addressing these limitations is necessary to validate and extend these findings for clinical application. Additionally, studies should explore the optimal concentrations and combinations of DMAHDM and/or MPC to balance the antimicrobial properties with mechanical strength without compromising surface hardness.

## 5. Conclusions

This research has demonstrated that incorporating DMAHDM and/or MPC into denture base materials significantly affects their mechanical properties, with notable differences observed between heat-polymerized (HP) and 3D-printed (3DP) resins where the HP exceeded those of the 3DP even after the improvement of the addition of the DMAHDM and/or MPC. This study highlighted that, while MPC generally maintains or improves flexural strength and surface hardness in HP materials, DMAHDM tends to reduce these properties, particularly at higher concentrations. In contrast, DMAHDM, especially when combined with MPC, showed potential in improving flexural strength and maintaining the elasticity of 3DP materials. Overall, this study provides valuable insights into developing more durable, antimicrobial denture materials, which could significantly improve oral health outcomes and quality of life for denture wearers. Further studies are planned to investigate the antimicrobial effects, biocompatibility, further mechanical properties, and the effect of accelerated aging on those properties.

## Figures and Tables

**Table 1:** Material specifications according to the manufacturers.

Tested Material	Abbreviation	Commercial Name	Company	City and Country	Ultimate Flexural Strength	Flexural Modulus	Residual Monomer
Heat Polymerized	HP	ProBase Hot	Ivocla Vivadent Inc.	Schaan, Liechtenstein	Comply with ISO 20795-1	Comply with ISO 20795-1	Comply with ISO 20795-1
3D-Printed	3DP	NextDent Denture 3D	NextDent	Soesterberg, The Netherlands	84 MPa	2383 MPa	<0.1

**Table 2:** Comparison of the mean flexural strength values between the six study groups in each of the tested materials (HP and 3DP).

Groups	Type of Material					
	HP			3DP		
	Mean Flexural Strength Value (SD)	F-Value	*p*-Value	Mean Flexural Strength Value (SD)	F-Value	*p*-Value
G1 (Control)	151.18 (22.67)	28.305	<0.0001	58.22 (6.01)	16.430	<0.0001
G2 (1.5% MPC)	145.25 (14.15)			43.15 (9.82)		
G3 (3% MPC)	147.94 (21.01)			44.87 (4.85)		
G4 (1.5% DMAHDM)	96.77 (16.62)			36.32 (4.13)		
G5 (3% DMAHDM)	62.67 (32.71)			61.02 (7.45)		
G6 (3% MPC + 1.5% DMAHDM)	106.83 (9.94)			62.76 (13.26)		

**Table 3:** Multiple comparisons of mean flexural strength values among the six study groups using the HP material.

Group	Comparison Groups	Mean Difference	*p*-Value
G1 (Control)	G2 (1.5% MPC) G3 (3% MPC) G4 (1.5% DMAHDM) G5 (3% DMAHDM) G6 (1.5% DMAHDM + 3% MPC)	5.924 3.240 54.410 88.510 44.346	0.990 0.999 <0.0001 <0.0001 <0.0001
G2 (1.5% MPC)	G3(3% MPC) G4(1.5% DMAHDM) G5(3% DMAHDM) G6 (1.5% DMAHDM + 3% MPC)	−2.684 48.485 82.585 38.422	1.000 <0.0001 <0.0001 0.004
G3 (3% MPC)	G4(1.5% DMAHDM) G5(3% DMAHDM) G6 (1.5% DMAHDM + 3% MPC)	51.170 85.270 41.106	<0.0001 <0.0001 0.001
G4 (1.5% DMAHDM)	G5(3% DMAHDM) G6 (1.5% DMAHDM + 3% MPC)	34.100 −10.063	0.008 0.903
G5 (3% DMAHDM)	G6 (1.5% DMAHDM + 3% MPC)	−44.163	<0.0001

**Table 4:** Multiple comparisons of mean flexural strength values among the six study groups using the 3DP material.

Group	Comparison Groups	Mean Difference	*p*-Value
G1 (Control)	G2 (1.5% MPC) G3 (3% MPC) G4 (1.5% DMAHDM) G5 (3% DMAHDM) G6 (1.5% DMAHDM + 3% MPC)	15.072 13.355 21.900 −2.797 −4.537	0.003 0.016 <0.0001 0.978 0.844
G2 (1.5% MPC)	G3(3% MPC) G4(1.5% DMAHDM) G5(3% DMAHDM) G6 (1.5% DMAHDM + 3% MPC)	−1.717 6.828 −17.870 −19.610	0.998 0.489 <0.001 <0.001
G3 (3% MPC)	G4(1.5% DMAHDM) G5(3% DMAHDM) G6 (1.5% DMAHDM + 3% MPC)	8.544 −16.153 −17.893	0.271 <0.001 <0.001
G4 (1.5% DMAHDM)	G5(3% DMAHDM) G6 (1.5% DMAHDM + 3% MPC)	−24.698 −26.438	<0.0001 <0.0001
G5 (3% DMAHDM)	G6 (1.5% DMAHDM + 3% MPC)	−1.740	0.997

**Table 5:** Comparison of the mean flexural strength values between the two study materials (HP and 3DP) within each of the six study groups.

Group	Mean Flexural Strength Values (SD)		Mean Difference	*p*-Value
	HP	3DP		
G1 (Control)	151.18 (22.67)	58.22 (6.01)	92.958	<0.0001
G2 (1.5% MPC)	145.25 (14.15)	43.15 (9.82)	102.105	<0.0001
G3 (3% MPC)	147.94 (21.01)	44.87 (4.85)	103.073	<0.0001
G4 (1.5% DMAHDM)	96.77 (16.62)	36.32 (4.14)	60.448	<0.0001
G5 (3% DMAHDM)	62.67 (32.71)	61.02 (7.45)	1.650	0.878
G6 (1.5% DMAHDM + 3% MPC)	106.83 (9.94)	62.76 (13.26)	44.073	<0.0001

**Table 6:** Comparison of the mean elastic modulus values between the six study groups in each of the tested materials (HP and 3DP).

Groups	Type of Material					
	HP			3DP		
	Mean E Modulus Value (SD)	F-Value	*p*-Value	Mean E. Modulus Value (SD)	F-Value	*p*-Value
G1 (Control)	5.42 (0.62)	27.741	<0.0001	0.80 (0.08)	24.99	<0.0001
G2 (1.5% MPC)	4.88 (0.26)			0.57 (0.12)		
G3 (3% MPC)	5.06 (0.52)			0.44 (0.07)		
G4 (1.5% DMAHDM)	3.34 (1.10)			0.49 (0.10)		
G5 (3% DMAHDM)	2.04 (1.18)			0.68 (0.13)		
G6 (3% MPC + 1.5% DMAHDM)	4.08 (0.21)			0.96 (0.17)		

**Table 7:** Multiple comparisons of mean elastic modulus values among the six study groups using the HP material.

Group	Comparison Groups	Mean Difference	*p*-Value
G1 (Control)	G2 (1.5% MPC) G3 (3% MPC) G4 (1.5% DMAHDM) G5 (3% DMAHDM) G6 (1.5% DMAHDM + 3% MPC)	0.531 0.360 2.080 3.380 1.331	0.654 0.896 <0.0001 <0.0001 0.005
G2 (1.5% MPC)	G3(3% MPC) G4(1.5% DMAHDM) G5(3% DMAHDM) G6 (1.5% DMAHDM + 3% MPC)	−0.171 1.549 2.849 0.800	0.996 0.001 <0.0001 0.242
G3 (3% MPC)	G4(1.5% DMAHDM) G5(3% DMAHDM) G6 (1.5% DMAHDM + 3% MPC)	1.720 3.020 0.971	<0.0001 <0.0001 0.078
G4 (1.5% DMAHDM)	G5(3% DMAHDM) G6 (1.5% DMAHDM + 3% MPC)	1.300 −0.749	0.005 0.283
G5 (3% DMAHDM)	G6 (1.5% DMAHDM + 3% MPC)	−2.049	<0.0001

**Table 8:** Multiple comparisons of mean elastic modulus values among the six study groups using the 3DP material.

Group	Comparison Groups	Mean Difference	*p*-Value
G1 (Control)	G2 (1.5% MPC) G3 (3% MPC) G4 (1.5% DMAHDM) G5 (3% DMAHDM) G6 (1.5% DMAHDM + 3% MPC)	0.230 0.355 0.311 0.120 −0.160	0.002 <0.0001 <0.0001 0.279 0.063
G2 (1.5% MPC)	G3(3% MPC) G4(1.5% DMAHDM) G5(3% DMAHDM) G6 (1.5% DMAHDM + 3% MPC)	0.125 0.081 −0.110 −0.390	0.234 0.694 0.343 <0.0001
G3 (3% MPC)	G4(1.5% DMAHDM) G5(3% DMAHDM) G6 (1.5% DMAHDM + 3% MPC)	−0.044 −0.235 −0.515	0.970 0.001 <0.0001
G4 (1.5% DMAHDM)	G5(3% DMAHDM) G6 (1.5% DMAHDM + 3% MPC)	−0.191 −0.471	0.015 <0.0001
G5 (3% DMAHDM)	G6 (1.5% DMAHDM + 3% MPC)	−0.280	0.997

**Table 9:** Comparison of the mean elastic modulus values between the two study materials (HP and 3DP) within each of the six study groups.

Group	Mean E. Modulus Values (SD)		Mean Difference	*p*-Value
	HP	3DP		
G1 (Control)	5.42 (0.62)	0.80 (0.08)	4.620	<0.0001
G2 (1.5% MPC)	4.89 (0.26)	0.57 (0.12)	4.319	<0.0001
G3 (3% MPC)	5.06 (0.52)	0.44 (0.07)	4.616	<0.0001
G4 (1.5% DMAHDM)	3.34 (1.09)	0.49 (0.10)	2.851	<0.0001
G5 (3% DMAHDM)	2.04 (1.18)	0.68 (0.13)	1.360	0.002
G6 (1.5% DMAHDM + 3% MPC)	4.08 (0.21)	0.96 (0.17)	3.129	<0.0001

**Table 10:** Comparison of the mean surface hardness values between the six study groups in each of the tested materials (HP and 3DP).

Groups	Type of Material					
	HP			3DP		
	Mean Surface Hardness Value (SD)	F-Value	*p*-Value	Mean Surface Hardness Value (SD)	F-Value	*p*-Value
G1 (Control)	18.05 (0.84)	18.519	<0.0001	13.57 (3.82)	27.440	<0.0001
G2 (1.5% MPC)	18.37 (0.310			5.78 (0.64)		
G3 (3% MPC)	15.36 (0.78)			5.34 (0.62)		
G4 (1.5% DMAHDM)	15.74 (1.45)			6.56 (0.94)		
G5 (3% DMAHDM)	10.07 (4.84)			5.29 (0.70)		
G6 (3% MPC + 1.5% DMAHDM)	14.86 (0.55)			7.14 (1.81)		

**Table 11:** Multiple comparisons of mean surface hardness values among the six study groups using the HP material.

Group	Comparison Groups	Mean Difference	*p*-Value
G1 (Control)	G2 (1.5% MPC) G3 (3% MPC) G4 (1.5% DMAHDM) G5 (3% DMAHDM) G6 (1.5% DMAHDM + 3% MPC)	−0.366 2.55 2.26 7.93 3.10	0.999 0.108 0.200 0.000 0.040
G2 (1.5% MPC)	G3 (3% MPC) G4 (1.5% DMAHDM) G5 (3% DMAHDM) G6 (1.5% DMAHDM + 3% MPC)	2.92 2.62 8.30 3.50	0.044 0.091 0.000 0.015
G3 (3% MPC)	G4 (1.5% DMAHDM) G5 (3% DMAHDM) G6 (1.5% DMAHDM + 3% MPC)	−0.029 5.38 0.58	1.000 0.000 0.992
G4 (1.5% DMAHDM)	G5 (3% DMAHDM) G6 (1.5% DMAHDM + 3% MPC)	5.67 0.87	0.000 0.956
G5 (3% DMAHDM)	G6 (1.5% DMAHDM + 3% MPC)	−4.79	0.000

**Table 12:** Multiple comparisons of mean surface hardness values among the six study groups using the 3DP material.

Group	Comparison Groups	Mean Difference	*p*-Value
G1 (Control)	G2 (1.5% MPC) G3 (3% MPC) G4 (1.5% DMAHDM) G5 (3% DMAHDM) G6 (1.5% DMAHDM + 3% MPC)	6.77 8.37 6.99 8.27 6.43	<0.0001 <0.0001 <0.0001 <0.0001 <0.0001
G2 (1.5% MPC)	G3 (3% MPC) G4 (1.5% DMAHDM) G5 (3% DMAHDM) G6 (1.5% DMAHDM + 3% MPC)	1.59 0.22 1.50 −0.35	0.512 1.000 0.578 0.999
G3 (3% MPC)	G4 (1.5% DMAHDM) G5 (3% DMAHDM) G6 (1.5% DMAHDM + 3% MPC)	−1.37 −0.09 −1.93	0.692 1.000 0.292
G4 (1.5% DMAHDM)	G5(3% DMAHDM) G6 (1.5% DMAHDM + 3% MPC)	1.27 −0.57	0.752 0.990
G5 (3% DMAHDM)	G6 (1.5% DMAHDM + 3% MPC)	−1.84	0.346

**Table 13:** Comparison of the mean surface hardness values between the two study materials (HP and 3DP) within each of the six study groups.

Group	Mean Surface Hardness Values (SD)		Mean Difference	*p*-Value
	HP	3DP		
G1 (Control)	18.00 (0.84)	13.56 (3.82)	4.44	0.002
G2 (1.5% MPC)	18.37 (0.31)	6.79(2.20)	11.58	<0.0001
G3 (3% MPC)	15.45(0.79)	5.20(0.75)	10.25	<0.0001
G4 (1.5% DMAHDM)	15.74 (1.45)	6.57 (0.94)	9.17	<0.0001
G5 (3% DMAHDM)	10.07 (4.84)	5.29 (0.70)	4.78	0.006
G6 (1.5% DMAHDM + 3% MPC)	14.86 (0.55)	7.14 (1.81)	7.72	<0.0001

## Data Availability

The data presented in this study are available on request from the corresponding author.
